# A Systematic Review of Physicians’ and Pharmacists’ Perspectives on Generic Drug Use: What are the Global Challenges?

**DOI:** 10.1007/s40258-014-0145-2

**Published:** 2015-05-12

**Authors:** Else-Lydia Toverud, Katrin Hartmann, Helle Håkonsen

**Affiliations:** Department of Social Pharmacy, School of Pharmacy, University of Oslo, P.O. Box 1068, Blindern, 0316 Oslo, Norway; Health Care Management, Bielefeld School of Public Health, University of Bielefeld, Bielefeld, Germany

## Abstract

**Background and Objectives:**

Generic substitution has been introduced in most countries in order to reduce costs and improve access to drugs. However, regulations and the generic drugs available vary between countries. It is the prescriber or dispenser of the drug who is the final decision maker. Nevertheless, physicians’ and pharmacists’ perceptions of generic drug use are not well documented to date. This study presents a systematic review of physicians’ and pharmacists’ perspectives on generic drug use worldwide.

**Methods:**

A systematic literature search was performed to retrieve all articles published between 2002 and 2012 regarding physicians’ and/or pharmacists’ experiences with generic drugs and generic substitution.

**Results:**

Of 1322 publications initially identified, 24 were eligible for inclusion. Overall, the studies revealed that physicians and pharmacists were aware of the cost-saving function of generic drugs and their role in improving global access to drugs. Nevertheless, marked differences were observed between countries when studying physicians’ and pharmacists’ perceptions of the available generic drugs. In less mature healthcare systems, large variations regarding, for example, control routines, bioequivalence requirements, and manufacturer standards were reported. A lack of reliable information and mistrust in the efficacy and quality were also mentioned by these participants. In the most developed healthcare systems, the participants trusted the quality of the generic drugs and did not hesitate to offer them to all patients regardless of socioeconomic status. In general, pharmacists seemed to have better knowledge of the concept of bioequivalence and generic drug aspects than physicians.

**Conclusions:**

The present study indicates that physicians and pharmacists are aware of the role of generic drugs in the improvement of global access to drugs. However, there are marked differences regarding how these health professionals view the quality of generic drugs depending on the maturity of their country’s healthcare system. This can be attributed to the fact that developed healthcare systems have more reliable public control routines for drugs in general as well as better bioequivalence requirements concerning generics in particular.

## Key Points for Decision Makers

**Table:** 

Generic drugs are generally seen as an important instrument for achieving better equity and access to drugs.
In mature healthcare systems, both pharmacists and physicians support the use of generic drugs and offer them to all patients regardless of socioeconomic status.
A lack of trust in manufacturers and the quality of the generic drugs affect how pharmacists and physicians consider generic drug use in less mature healthcare systems.

## Introduction

In order to reduce the growth in national healthcare spending, generic drugs are being increasingly used in most countries worldwide [1, 2]. Treatment of many patients, and in particular of those in developing countries, is now possible because of low-cost generic drugs [3]. However, drug control routines vary between countries, as do the number of drugs available. A brand name or reference drug can only be substituted by a generic drug when the latter contains the same active ingredient and strength as the reference drug, and is administered in the same dosage form. The difference in bioavailability between the two drugs should ideally lie within the therapeutic bioequivalence interval (the 90 % confidence interval of the ratio of a log-transformed test to reference mean area under the curve (AUC), which shows drug absorption, and maximum plasma level during drug absorption (*C*_max_) values falling within the range of 80–125 %) [4]. However, these regulations are not enforced by all governments. Further, the existence of several generic alternatives to a branded product leads to challenges for patients and healthcare personnel [5–7]. The switch from a brand name to a generic drug may prove more of a challenge for certain patient groups than others. For example, elderly patients and polypharmacy users can easily become confused, especially since the new product can differ in shape, taste, and colour [5, 7].

As early as 1968, the UK introduced compulsory generic prescribing as part of their national health system [8]. In the following decade, the first publications regarding experiences with generic substitution appeared in the USA [9, 10]. Since generic prescribing does not involve a switch from a specific brand prescribed by the physician to a product perceived as “cheaper” by the patient, it has been argued that generic prescribing might cause less confusion compared with generic substitution [11].

In 2012, Håkonsen and Toverud [2] published a review on patient perspectives on generic substitution. This review was exclusively based on studies from the developed world given the perceived limitations on the applicability of generic substitution in developing countries. Explanatory factors were high illiteracy rates, low educational levels, and limited access to healthcare, as well as large differences between rural and urban areas. It has been further suggested that patients in countries with mature healthcare systems are, in general, treated with medically adequate generic drugs [11]. Nevertheless, the challenges mentioned above can lead to reduced drug adherence or double dosing, and the issue of confusion can become even more severe if patients are treated by several physicians and attend different pharmacies [2]. Additionally, physicians and pharmacists respectively prescribing and dispensing drugs also face important challenges in relation to generic drug use. Exploring their perspectives and perceptions may thus increase the understanding of said challenges. Furthermore, by focusing on healthcare professionals with knowledge of their healthcare systems, it should be possible to obtain a broader international perspective of these challenges. Therefore, the aim of the present study was to systematically review physicians’ and pharmacists’ perspectives on generic drug use based on the available publications worldwide.

## Methods

A systematic literature search in MEDLINE (PubMed) and SciVerse Scirus (discontinued in 2014) was performed between July and December 2012 for peer-reviewed, original research articles regarding physicians’ and pharmacists’ experiences with and attitudes towards generic drugs and generic drug prescribing/substitution. The following terms were employed in the search strategy, using Boolean operators to refine the search (where possible, MeSH terms were applied): “generic drug”, “generic substitution”, “generic prescribing”, or “INN prescribing” combined with either “healthcare provider” and/or “healthcare professional” and/or “physician” and/or “pharmacist”.

**Fig. 1:**
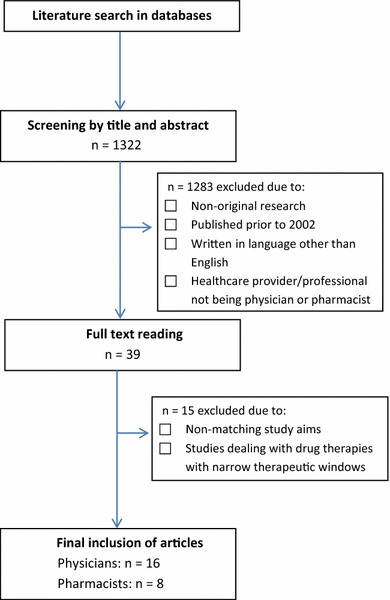
Flow chart of articles identified, screened, assessed for eligibility, and included in the review

Articles published in English from 2002 onwards were included. This publication time frame was chosen given that the process of generic substitution was generally established within this period in most countries. Publications based on prescription data were excluded. Figure 1 shows the initial number of identified articles, the assessment of eligible articles (according to the exclusion criteria), and the final number of included articles. Overall, 24 articles assessing physicians’ (*n* = 16) and pharmacists’ (*n* = 8) experiences and attitudes were included in the review. It is noteworthy that the previous review by Håkonsen and Toverud [2] on patients’ perspectives included a similar number of articles. Tables 1 and 2 provide an overview of the articles included, listed according to authorship and year of publication, country where the study was conducted, and the methodology applied.

**Table 1:** Articles regarding physicians’ perspectives included in the literature review (*n* = 16) in chronological order

References	Country	Method	Number of participants
Paraponaris et al. (2004) [12]	France	Postal survey, databases	600 (RR 55.8 %)
Kersnik and Peklar (2006) [13]	Slovenia	Postal survey	117 (RR 58.5 %)
De Run and Felix (2006) [14]	Malaysia	Personal interviews (semi-structured), postal survey	15 (^a^) 62 (^a^)
Hassali et al. (2006) [15]	Australia	Personal interviews (semi-structured)	10 (^a^)
Gossell-Williams (2007) [16]	Jamaica	E-postal/telephone/personal survey	60 (RR 60.0 %)
Heikkilä et al. (2007) [17]	Finland	Personal interviews (structured)	49 (^a^)
Polinski et al. (2008) [18]	Canada	Telephone survey	210 (RR 20.0 %)
Alghasham (2009) [19]	Saudi Arabia	Postal survey	772 (RR 85.8 %)
Tsiantou et al. (2009) [20]	Greece	Postal survey	1204 (RR 82.3 %)
Theodorou et al. (2009) [21]	Greece Cyprus	Postal survey	1204 (RR 82.3 %) 193 (RR 80.4 %)
Chua et al. (2010) [22]	Malaysia	Postal survey	87 (RR 26.8 %)
Shrank et al. (2011) [23]	USA	Web-based survey	506 (RR 18.3 %)
Jamshed et al. (2011) [24]	Pakistan	Personal interviews (semi-structured)	11 (^a^)
Jamshed et al. (2012) [25]	Pakistan	Self-administered questionnaires	289 (RR 71.3 %)
Fabiano et al. (2012) [26]	Italy	Web-based survey	303 (^a^)
Skinstad (2012) [27]	Norway	Telephone survey	91 (^a^)

*RR* response rate

^a^Response rate not applicable/unavailable

**Table 2:** Articles regarding pharmacists’ perspectives included in the literature review (*n* = 8) in chronological order

References	Country	Method	Number of participants
Allenet and Barry (2003) [28]	France	Postal survey	1000 (^a^)
Babar and Awaisu (2008) [29]	Malaysia	Postal survey	40 (^a^)
Gill et al. (2010) [30]	Australia, Finland, Italy	Personal interviews	45 (^a^)
Chong et al. (2011) [31]	Australia	Postal survey	500 (RR 16.4 %)
Babar et al. (2011) [32]	New Zealand	Postal survey	625 (RR 58.0 %)
Chong et al. (2011) [33]	Malaysia	Postal survey	219 (RR 15.4 %)
Olsson and Kälvemark Sporrong (2012) [34]	Sweden	Semi-structured interviews	16 (^a^)
Basak and Sathyanarayana (2012) [35]	India	Personal interviews (structured)	66 (^a^)

*RR* response rate

^a^Response rate not applicable/unavailable

## Results

### Physicians

Paraponaris et al. [12] conducted a study assessing the attitudes of 600 French general practitioners (GPs) regarding generic prescribing (Table 1) and found that 76 % of the respondents confirmed their willingness to do so. Gender, age, billing sector, length of practice, or additional degrees were not associated with this intention, nor were the socioeconomic background or unemployment rate of the local population. However, meeting with more than ten pharmaceutical sales representatives per week was a factor associated with reluctance for generic prescribing.

Another study of GP’s attitudes towards generic prescribing was carried out by Kersnik and Peklar [13] in Slovenia. This study included 200 GPs, representing 15 % of Slovenia’s GPs, who received a postal survey questionnaire and a prepaid return envelope. The majority of GPs responded that they usually met their patients’ demands or hospital consultant requests for branded products. Overall, 38 % did not consider the price when prescribing drugs, and 16 % felt that the pharmaceutical industry had a tremendous impact on their prescribing. Further, 90 % perceived generics to be as effective as branded drugs. Nevertheless, 25 % stated that they would only increase generic prescribing if additional clinical trials were presented.

De Run and Felix [14] initially interviewed 15 physicians from various public hospitals in one of Malaysia’s provinces using a semi-structured questionnaire. The insights obtained were used to develop a questionnaire answered by a further 62 physicians. The study explored physicians’ perceptions towards patented and generic drugs as well as factors influencing their prescribing decisions. The respondents viewed patented medicines as superior in quality, efficacy, and safety. Further, generic medicines were perceived as more affordable for the majority of the population, but lacking quality control and of uncertain efficacy. The physicians reported that the factors mostly affecting their prescribing decisions included their own experiences, evidence from the literature, their patients’ ability to afford the medication, and hospital policy.

In Hassali et al.’s [15] study performed in Melbourne, Australia, ten GPs were interviewed using a semi-structured questionnaire. The respondents had mixed attitudes towards generic prescribing—some viewed generics as equally effective as the innovator brand, but, when asked, no GP was aware of the acceptability criteria for bioequivalence of generic drugs. Further, they mentioned that they were concerned about patient confusion following substitution, and some felt that their role as a prescriber was threatened when pharmacists dispensed the generic drug.

Using the local telephone directory in Jamaica, Gossell-Williams [16] included and examined the attitudes of 100 physicians of various specialities (mostly GPs in private practices) towards generic substitution. Overall, 60 questionnaires were returned completed, 49 % of the physicians claimed that they prescribed generic drugs when cost was a significant factor for the patient, 29 % answered that they prescribed approximately equal numbers of the two drug categories, and 22 % said that they usually prescribed brand name drugs. Approximately half of the respondents felt that bioequivalent generics were therapeutically equivalent to branded drugs; 33 % stated that they could identify at least one case of clinical problems related to generic substitution over the previous year and mentioned that this would not have occurred with brand name drugs. The author concluded that more emphasis should be placed on improving physician confidence in the therapeutic equivalence of generics.

In Finland, Heikkilä et al. [17] explored how physicians (and customers) viewed the reform when generic substitution was introduced in 2003. The reform specifies that pharmacists are obliged to dispense the cheapest possible generic product as a substitute to prescribed medicines unless the physician has excluded substitution, which is only possible for medical reasons. In this study, 49 physicians (psychiatrists, geriatrists, internists, and GPs) were interviewed personally, and most believed that generic substitution was a good reform, mainly with regards to cost reduction. However, approximately half thought that not all interchangeable medicines were effective and safe.

Polinski et al. [18] assessed GPs’ opinions of generic substitution (introduced in 1994) and the Reference Drug Program (RDP) for the elderly (from 1995) in British Columbia, Canada. Of the 1050 GPs who were contacted, 210 agreed to be interviewed by telephone. Overall, the GPs rated the economic appropriateness of both generic substitution (87 %) and RDP (74 %) positively. However, they were less enthusiastic regarding the clinical appropriateness (70 % regarding generic substitution and 50 % regarding RDP). The most common concerns were whether drug switching and reduced adherence led to poorer health outcomes and whether the RDP could correctly identify therapeutically equivalent drugs.

Alghasham’s study [19] from Saudi Arabia assessed physicians’ perceptions and attitudes towards generic prescribing by sending a self-administered questionnaire to a random sample of 900 physicians from primary healthcare, hospitals, or private practice. The majority of the physicians (96 %) reported that they had adequate knowledge of the therapeutic value of the generic drugs they prescribed. Primary care physicians were significantly more likely to prescribe generically than hospital and private physicians (47, 31, and 22 %, respectively). However, only 16 % supported using generics (if available) in “all” clinical situations, whereas the majority supported generics in “most” cases. Further, 85 % were positive towards the government’s role in enforcing physicians to prescribe generic drugs. The physicians reported that representatives from generic drug companies were less likely to visit them than representatives from brand drug companies, and that they received significantly more drug samples from the brand name drug companies. An equal percentage of physicians “sometimes” felt pressured by patients to prescribe either brand drugs (41 %) or generic drugs (40 %).

Greek physicians’ perceptions and prescribing practices were studied by Tsiantou et al. [20] using a structured questionnaire with 25 semi-closed questions sent by mail to a random sample of 1463 physicians, stratified by gender, speciality, and geographical region. Overall, 75 % of physicians claimed that they were not affected by the sales representatives from drug companies and that Greek patients do not interfere with their prescribing, but often complain about the drug cost. When asked about quality, half of the respondents characterized the quality of generics as high or very high and claimed that implementation of an International Nonproprietary Name system was necessary. However, only 25 % prescribed generics.

Based on the study by Tsiantou et al. [20], Theodorou et al. [21] performed a comparison between Greek and Cypriot physicians. The previously developed postal questionnaire was sent to 240 physicians in Cyprus to add to the previously surveyed 1463 Greek physicians. Overall, 50 % of the Greek physicians and 60 % of those in Cyprus felt that the quality, safety, and effectiveness of generic drugs was “high” or “very high”. However, only 25 % of the Greek physicians said that they often prescribed a generic product instead of a branded one compared with 66 % of physicians in Cyprus. When the physicians in the two countries were questioned regarding their motivation to prescribe a generic drug, approximately 60 % indicated the patient’s out-of-pocket expenses for the drug were “important” or “highly important”.

Chua et al. [22] evaluated GPs’ knowledge and perceptions of generic medicines in Malaysia using a 23-item questionnaire sent by post to registered GPs. The majority of GPs (85 %) claimed that they actively prescribed generic drugs, but only 5 % correctly identified Malaysia’s National Pharmaceutical Control Bureau’s bioequivalence standard for generic products; there were misconceptions about the meaning of “bioequivalence”, “efficacy”, “safety”, and “manufacturing quality standards”. It appeared that, although the Malaysian GPs were largely prescribing generics, they still had concerns regarding the reliability and quality of such products. The GPs believed that a standard guideline on generic substitution, collaboration with pharmacists, patient education, and information on the safety and efficacy of generic medicines were necessary to ensure quality in the use of generics. The study revealed that the choice of drug depended on advertising and product incentives/bonuses offered by pharmaceutical companies, the patient’s socioeconomic background, and the credibility of manufacturers.

Shrank et al. [23] invited 2764 physicians from the USA to formulate their perceptions of generic medicines. Of the 839 doctors who completed the survey, 506 were found eligible and were included in the final study population. Of these, over 23 % were negative towards the efficacy of generic drugs and almost 50 % reported negative perceptions with regards to quality. Physicians older than 55 years of age were more than three times as negative to generics as younger doctors. The youngest physicians (under the age of 35) were significantly less likely to hold negative views regarding the quality and more likely to report a personal preference for generics or to recommend them to their family. Pharmaceutical companies were reported as the most common source of information about generic drugs (75 %).

Jamshed et al. [24] published a study including 11 physicians in Pakistan, where 80 % of the doctors both prescribe and dispense drugs, and bioequivalence studies are not required for generic substitution. The respondents were identified by snowballing, and the interviews were carried out using a semi-structured interview guide. It was revealed that the knowledge of dispensing doctors about generic medicines was sparse; for example, they confused the expression of generic substitution with generic prescribing. Further, mixed views and attitudes were identified towards generic medicines and the term bioequivalence was not understood. Some physicians considered generic medicines to be as safe and effective as any branded product when they were produced by high-quality national companies. The doctors admitted to being influenced by the pharmaceutical companies to preferentially prescribe their products; the acceptance of international trips to conferences and compensatory gifts (even cars) was admitted.

In a further study, Jamshed et al. [25] interviewed 289 randomly selected GPs with a questionnaire regarding their perceptions of and attitudes towards generic drugs. Close to 75 % of the respondents answered “correctly” regarding generics since they knew them to be “a copy of the brand name medicine” and/or “interchangeable with brand name medicines”. Overall, 55 % thought that generic medicines are therapeutically equivalent to branded drugs, whereas 59 % said that generics are less safe, and 58 % were of the opinion that only local reputable manufacturers produce safe generic medicines. Concerning brand name products, physicians stated that they were of better quality than generics (59 %), that they were required to meet higher safety standards (77 %), and that they had fewer side effects (66 %). The authors concluded that gaps in knowledge of generic medicines had been identified among their participants.

In Italy, Fabiano et al. [26] conducted a nationwide web-based survey aiming to evaluate the knowledge of generic medicines and prescribing habits of family paediatricians. The hypothesis was that Italian GPs and family paediatricians were less confident in prescribing generics than physicians elsewhere and, therefore, that generic drug prescribing would be low. In total, 303 family practitioners filled out the online questionnaire. The majority believed that the efficacy of generic medicines was sufficient (34 %) or good (45 %), and 37 and 33 % of them declared themselves to have a sufficient or fairly good knowledge of generic medicines, respectively. However, only 14 % stated that as much as half of their patients were treated with generics. The major issues preventing generic prescribing were identified to be the widespread scepticism about the reliability of bioequivalence tests and the safety of switching from branded to generic equivalents. The authors concluded that more information regarding generic drugs and further research in the field of paediatric pharmacology were required to increase generic drug prescribing among Italian family paediatricians.

Using a structured questionnaire, Skinstad [27] performed telephone interviews with 91 GPs from all counties in Norway regarding their attitudes and experiences with generic substitution. In Norway, it is obligatory for the pharmacist to perform generic substitution if the physician has not actively excluded substitution for medical reasons on the prescription, unless the patient denies substitution and pays the price difference between the generic and the brand product. Overall, 75 % of doctors were positive toward the system despite complaining that it was time consuming. They also feared impaired health outcomes since they were worried about poor drug adherence caused by confusion and anxiety. Only half of the doctors reported that they prescribed generics to some or a high degree. A key reason for brand name prescribing was their memory of the brand name and the confidence that the pharmacist would take care of the substitution.

### Pharmacists

Allenet and Berry [28] assessed the opinions and behaviour of community pharmacists in France towards generic substitution (Table 2). Since 1999, the pharmacists have had the right to switch from branded to generic medicines unless the prescriber has specified otherwise. A structured questionnaire was sent by mail to be filled out by the pharmacy owner. Most respondents (91 %) agreed that the pharmacists’ right to substitute is “a good thing”. Less than half of the study population (43 %) reported that they systematically offered the patient a generic drug, whereas 55 % claimed to target specific populations for substitution. Overall, 80 % felt that the substitution system improved pharmacists’ influence within the healthcare system, but at the same time, 70 % stated that substitution was difficult to implement. Only 51 % considered the financial compensation for the sale of generic drugs to be satisfactory, and 85 % stated that training was necessary. Nevertheless, the majority (86 %) had positive attitudes towards generic prescribing.

Babar and Awaisu [29] evaluated community pharmacists’ perceptions and practices on generic drugs in the Malaysian peninsula. Of the pharmacists surveyed, 47 % recommended original brands over generics, whereas the opposite was stated by another 47 %. Further, 45 % had “high” or “very high” confidence in the Malaysian generic approval system regarding bioequivalence, and as much as 93 % of the respondents were confident that the generic drugs they dispensed had been subjected to bioequivalence control before approval. The participants said that they always considered affordability for customers first, and 85 % felt that the customer usually accepted their recommendation for generic drugs. However, 62 % said that compulsory generic substitution should not be implemented in Malaysia since the situation with generic drugs remained unclear.

Gill et al. [30] studied how pharmacists (and customers) experienced generic substitution in Finland as compared with Australia and Italy by interviewing 15 pharmacists from each country. In Finland, where generic substitution is generally accepted, the pharmacists were concerned about customer confusion following substitution. A pharmacist stated: “don’t talk about substitution to old customers who have many challenges already, or those who have dementia”, whereas another stated: “I have to offer the generics even if the customer is not listening.” The Australian pharmacists reported that it took time to instruct “resistant customers”, especially polypharmacy users, patients with mental illness, and those with dementia. The Italian and Australian pharmacists experienced frustration when the customer did not believe that the generic was equivalent to the branded medicine. The respondents also reported that physicians act as a significant barrier; the Australian pharmacists, for instance, remarked that 50 % of the patients requested discussion with their doctor before they would accept generic substitution. In general, pharmacists in all three countries felt that it was a professional challenge to educate customers about the generic substitution system.

Chong et al. [31] evaluated the Australian community pharmacists’ rate of generic substitution, patient acceptance of the substitution, and the cost saving achieved for patients. A national stratified sample of 500 pharmacies was randomly selected, and the first 25 original prescription items that were eligible for generic substitution dispensed during 1 working day were collected from each pharmacy. It was found that the pharmacists recommended generics for 96 % of the items that were eligible for substitution. Further, the recommendation rate was significantly higher in urban areas compared with remote areas, whereas the opposite was found for patient acceptance. A significant difference between patient acceptance for chronic diseases (72 %) and acute conditions (82 %) was also noted. Through acceptance of substitution, the patients’ medicine expenditure was reduced by approximately 21 %. Finally, the authors concluded that patient acceptance required further improvement.

Babar et al. [32] surveyed pharmacists’ (*n* = 625) views, knowledge, and perception of generic medicines in New Zealand. Only 30 % of pharmacists correctly identified the properties of generic medicines (namely safety, effectiveness, quality, and cost). Those who had been in practice less than 5 years had a better understanding of the substitution system than those who had been in practice for 5 years or more. As much as 65 % believed that original brand products were of better quality, and 70 % perceived no difference in safety between original brands and generics. However, 16 % were against generics produced in emerging markets. The pharmacists’ perception was reported to be affected by consumer preferences or demand (76 %), professional judgment (72 %), and the manufacturer’s reputation (45 %). Furthermore, 56 % thought that generic medicines had no impact on adherence and 76 % stated that generic medicines are cost effective for the New Zealand healthcare system. Nevertheless, only 3 % supported more frequent prescribing of generic medicines and 84 % were worried about a “reduced profit” when dispensing generic drugs.

Chong et al. [33] evaluated community pharmacists’ views on generic drugs in Malaysia. Half of the pharmacists agreed that all products approved to be bioequivalent could be considered therapeutically equivalent to the reference drug (brand name drug), and 76 % indicated that generic substitution of drugs with narrow therapeutic windows was inappropriate. The majority of the pharmacists (85 %) stated that a generic medicine must contain the same amount of the active ingredient as the original brand, and 72 % stated that it must be in the same dosage form. Approximately 21 % thought that generic medicines, in general, were of inferior quality compared with the original, and a minority also thought that generic drugs had more side effects. They further reported that customers showed a high degree of mistrust when the manufacturer was unknown and deemed it difficult to explain that a drug with a different shape and colour can have the same efficacy as the drug previously used.

Olsson and Kälvemark Sporrong [34] interviewed 16 Swedish pharmacists using a semi-structured interview guide and found that most pharmacists were in favour of generic substitution because of the economic benefits. However, they felt that they lacked education regarding both the substitution system and generic drugs in general. Most considered generics to be equivalent with and as effective as the original product. In contrast to these statements, some pharmacists were confused since many patients reported a lack of effect as well as new side effects. The pharmacists felt that a reason for the mistrust could be that generics could have different tablet coating, lack calendar packaging, and not always be packed in “the same exclusive way” as the brand products. The pharmacists reported that more time-consuming work with the patients was required for generic substitution and that discussions regarding generics had taken over the patient–pharmacist relationship. Nevertheless, the pharmacists were positive about more generic prescribing.

In India, Basak and Sathyanarayana [35] carried out a survey to evaluate community pharmacists’ and drug retailers’ knowledge on and perceptions of generic drugs. The study was conducted in 39 randomly selected private pharmacies. Among the 66 respondents, 39 (59 %) were drug retailers. Overall, 32 % did not know what generic medicines were and 64 % believed that generic medicines could be considered therapeutically equivalent with branded drugs. However, 30 % believed that generics were of inferior quality to the branded drugs. A higher level of education had a significant correlation with knowledge of generic medicines (*P* < 0.01). Finally, the majority of the respondents (80 %) were against generic substitution even if the branded drug was not available.

## Discussion

The present study shows that physicians and pharmacists have acknowledged strategies for generic drug use as an attempt to curtail increasing drug expenditure. However, their perceptions vary according to the maturity of their country’s healthcare systems (Table 3). The discussion section below is divided thematically on the basis of the topics discussed in the various articles.

**Table 3:**
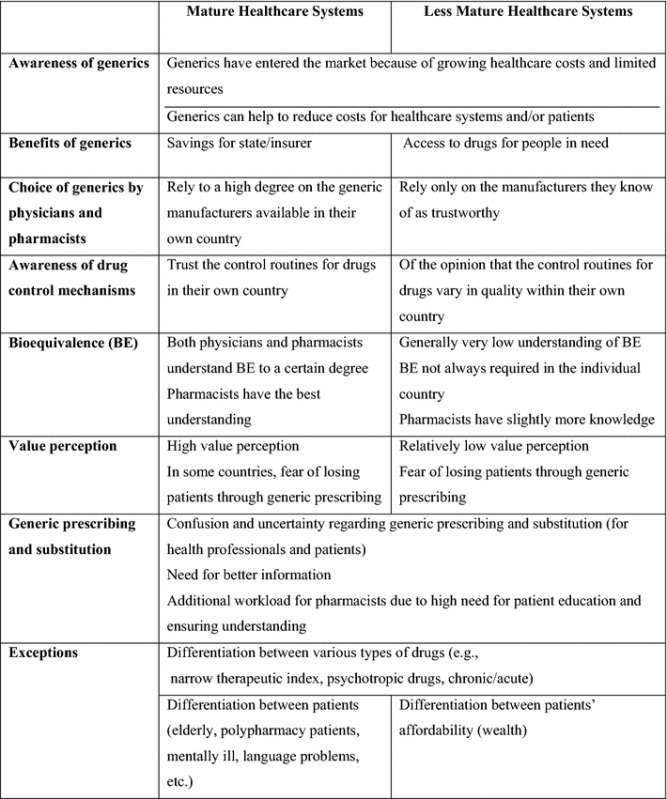
Summary of physicians’ and pharmacist’s perceptions of generic drugs as reported in the literature

### The Manufacturer

It is well known that, in most countries, generic drugs can be manufactured both nationally and internationally. A key observation of the present review is the marked variation in the level of trust health professionals had in generic drug companies within countries. For instance, in northern Europe, the health professionals felt confident about the generic drugs available in the market [17, 27, 34], and in Australia, the majority of pharmacists were found to recommend generics [31]. However, it was reported that physicians in southern Europe depended on information provided by the brand drug manufacturers to a greater degree than physicians in northern Europe [12, 13, 20]. In countries with less mature healthcare systems, both physicians and pharmacists were highly concerned about the manufacturing sources of generic drugs and the companies’ trustworthiness [22, 24, 25, 29, 30, 32, 33]. In some emerging healthcare systems, non-product-related promotion was still much more accepted than the dissemination of product- or therapy-related information [24].

### Control Routines and Bioequivalence Requirements

Inferior regulatory and control processes for drugs combined with price pressures increase the risk of counterfeit drug marketing. The present review found that both healthcare personnel categories assessed appeared to have an understanding of the reliability of the control routines in their own country and adapted their level of trust in the system accordingly [12, 19, 23, 25–27, 34]. A general marked variation between countries with mature healthcare systems and those with developing healthcare systems was observed once again. It was further elucidated that, even if controls were in place, the requirements for proof of bioequivalence were not necessarily comprehensive [16, 19, 24, 25, 29, 35]. In general, pharmacists appeared to have a better knowledge on the concept of bioequivalence than physicians, particularly in the case of elderly doctors, in whom the lack of familiarity with the importance of bioequivalence for drug interchangeability was obvious [16, 22, 24, 25].

### Therapeutic Window

Although articles related to specific drugs were not included in the present review, it was found that many physicians and pharmacists differentiated between drugs when considering generic substitution. They were convinced that generic substitution should not occur for certain branded drugs [17] and were especially sensitive to drugs with a narrow therapeutic window [33].

### Which Patients Should be Offered Generics?

In regions like the USA, Australia, and northern Europe, generics were, in general, offered to patients from all socioeconomic backgrounds [17, 27, 30, 31, 34]. However, the physicians and pharmacists appeared to be worried about certain patient categories for whom switching to generics or switching between different generics should not be recommended. Examples of patient categories in which confusion may arise included elderly patients, polypharmacy users, mentally ill patients, and patients who do not speak the local language [15, 27, 30, 34]. In contrast, in some countries in southern Europe and in some countries with an early-stage healthcare system, both physicians and pharmacists raised concerns regarding the loss of patients/customers if generics were prescribed or suggested, as well as affordability for the patient [14, 16, 21, 22, 28, 29].

### The Quality of the Drug

In the present review, pharmacists from the Nordic countries focused on the physical and packaging differences, such as tablet coating and calendar packaging, between the branded and generic drugs [34]. As mentioned above, health professionals in northern European countries had a high level of confidence that a generic drug was identical to the branded analogue regarding the active ingredient, dosage, and therapeutic effects [17, 27, 34]. These health professionals expressed a greater concern for patient adherence, which might be lowered by misunderstanding and anxiety [15, 27]. In most other countries, the two groups of health professionals were more worried about the efficacy of the drug. In less mature healthcare systems, generics were believed to have a lower or uncertain efficacy and were often looked upon as being inferior in quality on the basis of negative experiences, attitudes, and perceptions [12, 14–16, 18, 20–26, 29, 32, 33, 35].

### More Information Needed

An overarching concern was the urgent need for more reliable information on generic drugs and generic prescribing/substitution [19, 22, 24–26, 31]. Some felt that this should be included in medical or pharmaceutical training curricula, since all practising physicians and pharmacists were increasingly being confronted with the need to prescribe or dispense these medications [22, 34]. The available data indicated that pharmacists are currently better informed, which is not surprising given that their frequent contact with patients/customers requires an explanation of the nature of generic drugs [15, 17, 28, 30, 31, 34].

## Limitations

A limitation of the present study was the exclusion of articles written in languages other than English.

## Conclusions

The present study shows that physicians and pharmacists are aware of the potential of generic drugs in the improvement of global access to drugs. However, there are marked differences in how pharmacists and physicians consider the quality of generic drugs depending on the maturity of the healthcare system in their own country. This can be attributed to the fact that developed healthcare systems have more reliable public control routines for drugs in general as well as better bioequivalence requirements concerning generics in particular.
